# Element contamination of the Orange-Vaal River basin, South Africa: a One Health approach

**DOI:** 10.1007/s11356-024-32932-8

**Published:** 2024-04-09

**Authors:** Johannes Hendrik Erasmus, Marliese Truter, Nico Jacobus Smit, Milen Nachev, Bernd Sures, Victor Wepener

**Affiliations:** 1 Water Research Group, Unit for Environmental Sciences and Management, North-West University, Potchefstroom, 2520 South Africa; 2 South African Institute for Aquatic Biodiversity (NRF-SAIAB), Makhanda, South Africa; 3 Department of Aquatic Ecology and Centre for Water and Environmental Research, University of Duisburg-Essen, 45141 Essen, Germany; 4 Research Center One Health Ruhr, Research Alliance Ruhr, University of Duisburg-Essen, Essen, Germany

**Keywords:** *Clarias gariepinus*, Bioaccumulation, Chronic human health risks, Metal contamination, Mining and industrial activities, Multi-stressor environment

## Abstract

Numerous low-income groups and rural communities depend on fish as an inexpensive protein source worldwide, especially in developing countries. These communities are constantly exposed to various pollutants when they frequently consume polluted fish. The largest river basin in South Africa is the Orange-Vaal River basin, and several anthropogenic impacts, especially gold mining activities and industrial and urban effluents, affect this basin. The Department of Environment, Forestry and Fisheries in South Africa has approved the much-anticipated National Freshwater (Inland) Wild Capture Fisheries Policy in 2021. The aims of this study were (1) to analyze element concentrations in the widely distributed *Clarias gariepinus* from six sites from the Orange-Vaal River basin and (2) to determine the carcinogenic and non-carcinogenic human health risks associated with fish consumption. The bioaccumulation of eight potentially toxic elements (As, Cd, Cr, Cu, Hg, Ni, Pb, Zn) was assessed in *C. gariepinus* from sites with different anthropogenic sources. The human health risks were determined to assess the potential risks posed by consuming contaminated *C. gariepinus* from these sites. Carcinogenic health risks were associated with fish consumption, where it ranged between 21 and 75 out of 10,000 people having the probability to develop cancer from As exposure. The cancer risk between the sites ranged between 1 and 7 out of 10,000 people to developing cancer from Cr exposure. A high probability of adverse non-carcinogenic health risks is expected if the hazard quotient (HQ) is higher than one. The HQ in *C. gariepinus* from the six sites ranged between 1.5 and 5.6 for As, while for Hg, it was between 1.8 and 5.1. These results highlight the need for monitoring programs of toxic pollutants in major river systems and impoundments in South Africa, especially with the new fisheries policy, as there are possible human health risks associated with the consumption of contaminated fish.

**Graphical Abstract:**

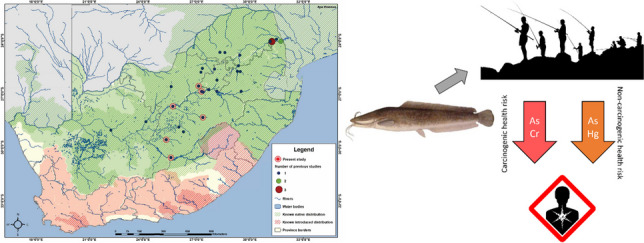

## Introduction

A major challenge in South Africa is that the river systems and impoundments are under various threats including intensive mining, industrial and agricultural activities, and urban effluent, as well as untreated and treated sewage, resulting in contaminated aquatic ecosystems (DWS 2019). An Ecological Condition Index (ECI) was developed to assess the overall ecological health of aquatic systems, where a score of 100 refers to natural or pristine conditions, while 0 indicates critically or severely modified conditions, and natural ecosystem functions have totally been lost (DWS 2019). The state of rivers in South Africa has decreased from an ECI score of 83 in 1999 to 72 in 2011, with lowland rivers being the most impacted compared to other river types (Stats SA 2017; DWS 2019). Although some river systems do not drain these areas of intensive anthropogenic impacts, most of South Africa’s rivers drain naturally high metal–rich geology, and the addition of element concentrations of anthropogenic stressors in these rivers poses a major threat (Wepener et al. 2011; Erasmus et al. 2022b). The Orange River basin is South Africa’s largest basin, having a catchment area of 973,000 km^2^, covering 77% of the land surface of South Africa (van As et al. 2013). The Orange River originates in the highlands of Lesotho and meanders through South Africa for 2200 km before draining into the Atlantic Ocean. It consists of two major tributaries, the Caledon River draining the highlands of Lesotho (with minimal anthropogenic activities as agriculture is the main land-use activity), and one of the most polluted rivers, the Vaal River, which drains the inland plateau (with extensive industrial, mining, agricultural, and urban activities) (Wepener et al. 2011).

Aquatic ecosystems provide several ecosystem services, and one of the main services is utilizing fish as a food source. Fish play an important role as a food source globally (Sayer and Cassman 2013), but low-income households and communities in developing countries are more dependent on fish as an inexpensive protein source that can be harvested locally from rivers and impoundments (Kawarazuka and Béné 2011; Erasmus et al. 2022b). In South Africa, the Department of Forestry, Fisheries and the Environment (DFFE) has approved the much-anticipated National Freshwater (Inland) Wild Capture Fisheries Policy in 2021 that aims for a sustainable development approach to the use of natural freshwater resources for the benefit of all citizens (DFFE 2021). Thus, small-scale fisheries and fishing for subsistence and livelihood purposes by local and rural communities are approved and encouraged. Weyl et al. (2021) identified the lack of knowledge on the influence of environmental pollutants on ecosystem health and how this relates to fish health, which in turn influences human health, as a major concern.

There is an increased awareness that complex environmental issues need to be addressed in an interdisciplinary manner. The One Health (OH) concept was originally formulated as an interdisciplinary approach to ensure optimal health for animals, the environment, and people through collaborative actions between health and environmental sciences (Mackenzie et al. 2013; Selbach et al. 2022). In the past, OH mainly focused on human and veterinary health and although the concept has expanded beyond its original narrow focus (mainly zoonotic diseases), the focus on environmental health remains an under-represented or neglected component (Essack 2018). From the 6023 articles published on the OH concept from 2000 until 2021, only 616 articles (10%) focused on the health of aquatic ecosystems (Selbach et al. 2022). Of the 10% of articles that focused on aquatic ecosystems, most of them only discussed the response to individual public health emergencies, while the important and inseparable links between animal, environmental, and human health are not acknowledged (Essack 2018).

In the last decade, more studies acknowledged the use of fish as bioindicator species in linking animal health with human health, especially as food source in a One Health approach (Musoke et al. 2016; Murtaugh et al. 2017; Prata et al. 2021; Vergis et al. 2021; Multisanti et al. 2022). The use of fish and their attributes has proven to be successful in assessing the integrity and ecological status of aquatic ecosystems and identifying impacts affecting these systems for more than 25 years (Barbour et al. 1999). An ideal fish species to use in South Africa is the sharptooth catfish, *Clarias gariepinus* due to the following: (i) large natural and introduced distribution (thus occurring in most South African river systems); (ii) wide tolerance to extreme environmental conditions; (iii) they are mostly associated with benthic feeding, are omnivorous and opportunistic feeders, and occupy high tropic levels; and (iv) most importantly is a valued food source for humans (Skelton, 2001; Picker and Griffiths 2011; Musa et al. 2017; Froese and Pauly 2023). Their association with sediment and the feeding habits of *C. gariepinus* make them ideal representatives of element bioaccumulation (especially toxic elements) in aquatic systems, and with the fact that some elements can biomagnify through food webs, they can pose human health risks to communities chronically exposed to these toxic elements when they frequently consume polluted fish (Erasmus et al. 2022b).

Therefore, the aims of the present study were to (i) establish the element concentrations in *C. gariepinus* from six sites in the Orange-Vaal River basin and representing the environmental conditions of these sites, (ii) determine the carcinogenic and non-carcinogenic human health risks associated with the consumption of this fish species, and (iii) integrate the important link between animal, environmental, and human health using the OH concept.

## Materials and methods

### Study area

**Fig. 1:**
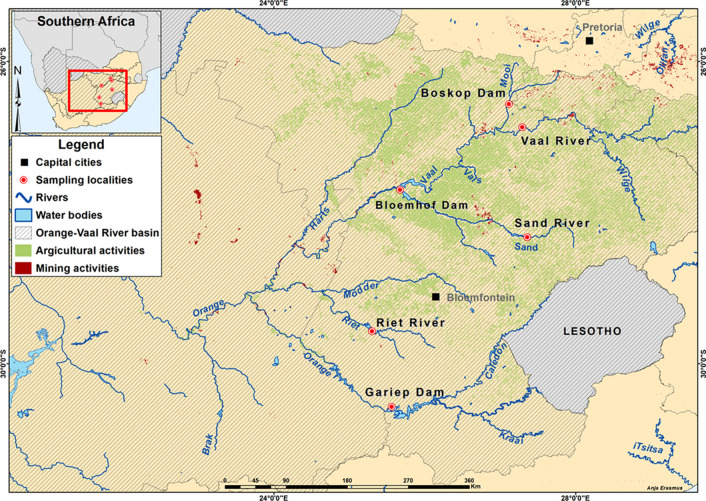
Map of the study area, indicating the Orange-Vaal River basin (striped area), major river systems within the basin, and agricultural (green overlay) and mining activities (red overlay), as well as the six sampling sites

Six sites were selected within the Orange-Vaal River basin: Boskop Dam, Mooi River (draining extensive formal gold mining, as well as artisanal gold mining activities), Bloemhof Dam (a major impoundment in the Vaal River at the confluence of the Sand and Vaal Rivers, draining several gold mining, agricultural, and urban activities), Gariep Dam (a major impoundment in the Orange River after the confluence of the Caledon River, draining mostly agricultural activities), Riet River (draining minimal anthropogenic activities), Sand River (draining several agricultural, gold mining, and urban activities), and the Vaal River (draining extensive agricultural, industrial, mining, and urban activities). These sites were selected as representative sites for the Orange-Vaal River basin (Fig. 1).

### Sample collection

Field surveys were carried out between March 2018 and November 2020 at six sites within the Orange-Vaal River basin. The necessary ethical clearance (NWU-00159-18-A5) and permits from the two different provinces (Free State (JM 4066/2018); North West (HO 20/02/18-057 NW, NW 8065/03/2019)) for collecting and euthanizing fish were obtained prior to sampling. The samples of *Clarias gariepinus* were collected using fyke nets and multi-filament gill nets as described in Erasmus et al. (2019), as well as longline fishing techniques from Boskop Dam (*n* = *15*), Bloemhof Dam (*n* = 10), Gariep Dam (*n* = 15), Riet River (*n* = *12*), Sand River (*n* = 12), and Vaal River (*n* =18). The fish were euthanized according to accepted protocols (SOP, NWU-00267-17-A5) by cervical transection and pithing; subsequently, white axial muscle samples (± 20 g wet weight (w.w.)) were collected from each individual and stored in acid-washed polypropylene containers at − 20 °C until trace element analyses.

### Trace element analysis

Muscle tissues were freeze-dried (FreeZone 6, Labconco) for 96 h and the mean moisture content was determined as 68%. Samples were homogenized and approximately 200-mg sample was digested in 7.5 ml 65% HNO_3_ (sub-boiled, Merck) and 2.5 ml 37% HCl (supra pure quality, Merck) acid using a microwave digestion system (Ethos Easy, Milestone) following the methods described in Erasmus et al. (2022a). After digestion, the sample solution was transferred to a 50-ml volumetric flask and brought to volume with 1% HNO_3_, whereafter it was transferred to pre-cleaned polypropylene containers for storage at room temperature until trace element analysis.

The concentrations of As, Cd, Cu, Ni, Pb, and Zn in muscle tissue samples were determined by using quadrupole inductively coupled plasma mass spectroscopy (ICP-MS) (Elan 6000, PerkinElmer). Concentrations of Cr were determined using a graphite furnace atomic absorption spectrometer (GF-AAS) (AAnalyst 600, PerkinElmer) equipped with Zeeman-effect background correction, while concentrations of Hg were determined by using a Flow Injection Mercury System (FIMS 400, PerkinElmer). The concentrations of all trace elements were expressed in milligrams per kilogram dry weight (d.w.). Instrument operational settings, calibration methods, sample preparation, and quality assurance for ICP-MS and GF-AAS were as described in Erasmus et al. (2020a), while for FIMS as described in Erasmus et al. (2022a).

### Quality control and quality assurance

Quality control (QC) and quality assurance (QA) were ensured by analyzing quality standards (QS) after every 20 samples, while certified reference material (CRM) DORM-4 (fish protein certified reference material, National Research Council, Canada) was analyzed and treated in the same manner as samples. Three blank samples per digestion run were prepared in the same way as the samples and CRMs, resulting in a total of 12 blank samples. Recovery rates for eight CRM samples were within 20% of the certified range (Table 1) (Erasmus et al. 2020a; Díaz-Morales et al. 2021). Each sample (blanks, CRM, and muscle tissue) was analyzed in triplicate where the mean concentration was used, while the percentage relative standard deviation (%RSD) was always less than 10%. The limit of detection and limit of quantification were calculated as three and nine times the standard deviation of the blank measurements based on the mean muscle weight of 212 mg, respectively (Table 1).

**Table 1:** Recovery rates (%), limit of detection (LOD), and limit of quantification (LOQ) (mg/kg d.w.) of the trace elements of interest for fish protein certified reference material (DORM-4)

Element	Recovery (%)	LOD (mg/kg)	LOQ (mg/kg)
As	116	0.00316	0.00947
Cd	101	0.00004	0.00014
Cr	98	0.19084	0.57252
Cu	85	0.00156	0.00469
Hg	97	0.00394	0.01183
Ni	93	0.00012	0.00037
Pb	87	0.00108	0.00325
Zn	94	0.01495	0.04486

### Human health risk assessment

Various international organizations have published instructions and standards on the estimation of potential risks to human health, through the consumption of fish, which is polluted with several environmental contaminants, especially toxic elements (USEPA 2016). In the present study, both carcinogenic and non-carcinogenic human health risk assessments were calculated using the methods described by the US Environmental Protection Agency (USEPA 2000; USEPA 2005), while adapting it to represent more realistic conditions in South Africa as in Heath et al. (2004), while all of the equations were used as described in Erasmus et al. (2022b).

### Statistical data analyses

Normality and homogeneity of variance were tested using D’Agostino and Pearson omnibus normality test and Shapiro-Wilk normality test, respectively. In order to test for significant differences in element concentrations between sites, a one-way analysis of variance (ANOVA) with Tukey’s multiple comparison test was used. The level of significance was set at *p* < 0.05. A principle component analysis (PCA) was constructed to assess the spatial patterns associated with the element concentrations in *C. gariepinus* muscle, while the biometric data (total length (TL), weight, and sex) were overlayed as supplementary data. To normalize the data used for the PCA, log transformation (*y* = log (*x* + 1)) was applied.

## Results

### Element bioaccumulation in *Clarias gariepinus*

**Fig. 2:**
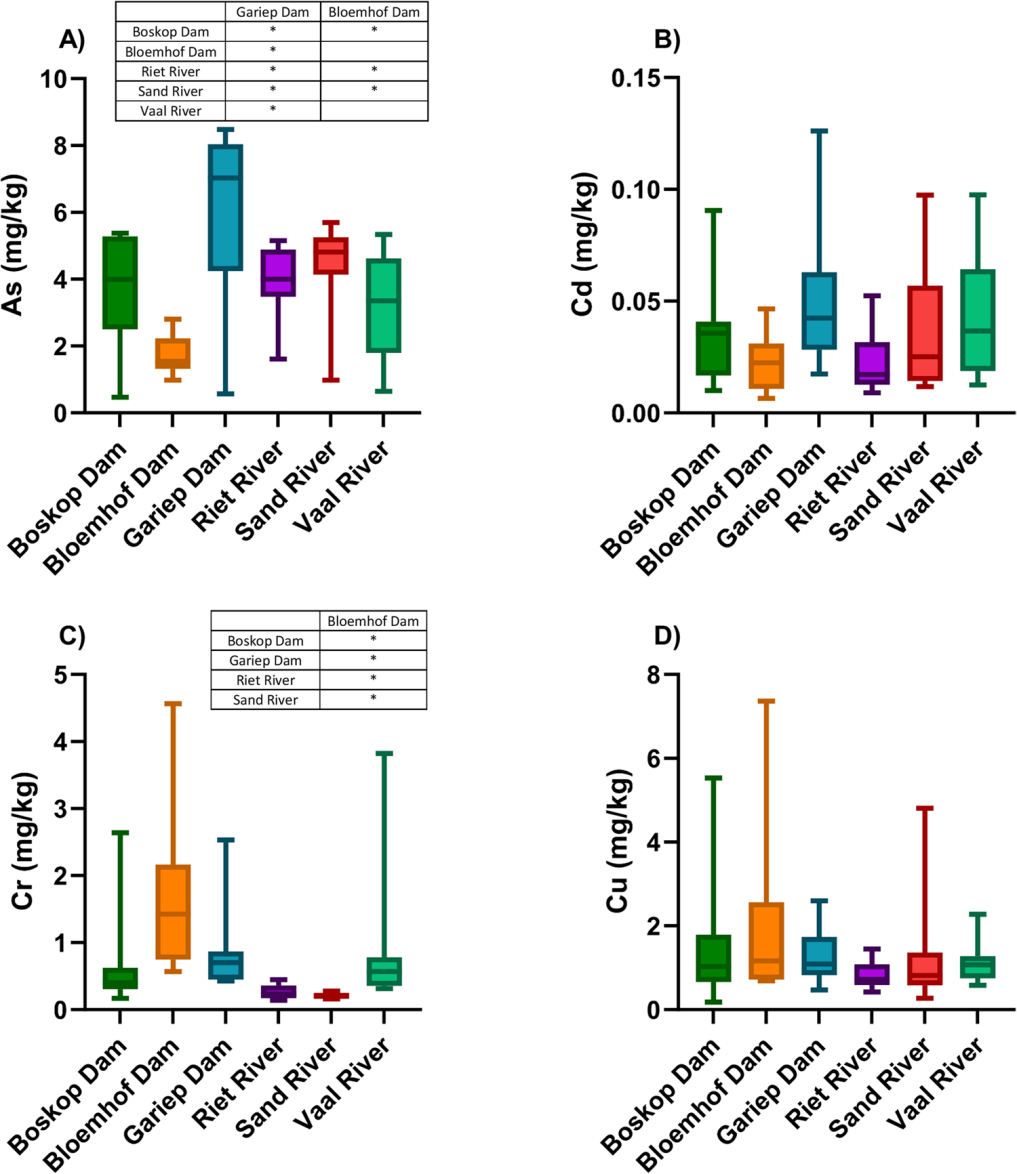
Box and whisker plots of the concentrations (mg/kg d.w.) of **A** As, **B** Cd, **C** Cr, and **D** Cu in *Clarias gariepinus* from the six sampling sites in the Orange-Vaal River basin. Mean concentrations are indicated with a solid line and the whiskers indicates the 5^th^ and 95^th^ percentiles. Significant differences (*p* < 0.05) between sites are indicated in table format by means of an asterisk

The concentrations of As in *C. gariepinus* were significantly (*p* < 0.05) higher at the Gariep Dam compared to all the other sites, while Bloemhof Dam had the lowest As concentrations and was significantly (*p* < 0.05) lower compared to Boskop Dam, Riet River, and the Sand River (Fig. 2A). Although not significant, Gariep Dam’s *C. gariepinus* also had the highest Cd concentrations compared to all other sites (Fig. 2B). Bloemhof Dam’s *C. gariepinus* had significantly (*p* < 0.05) higher Cr concentrations than all the other sites (Fig. 2C), while the concentrations of Cu were also the highest in *C. gariepinus* from Bloemhof Dam (Fig. 2D).

**Fig. 3:**
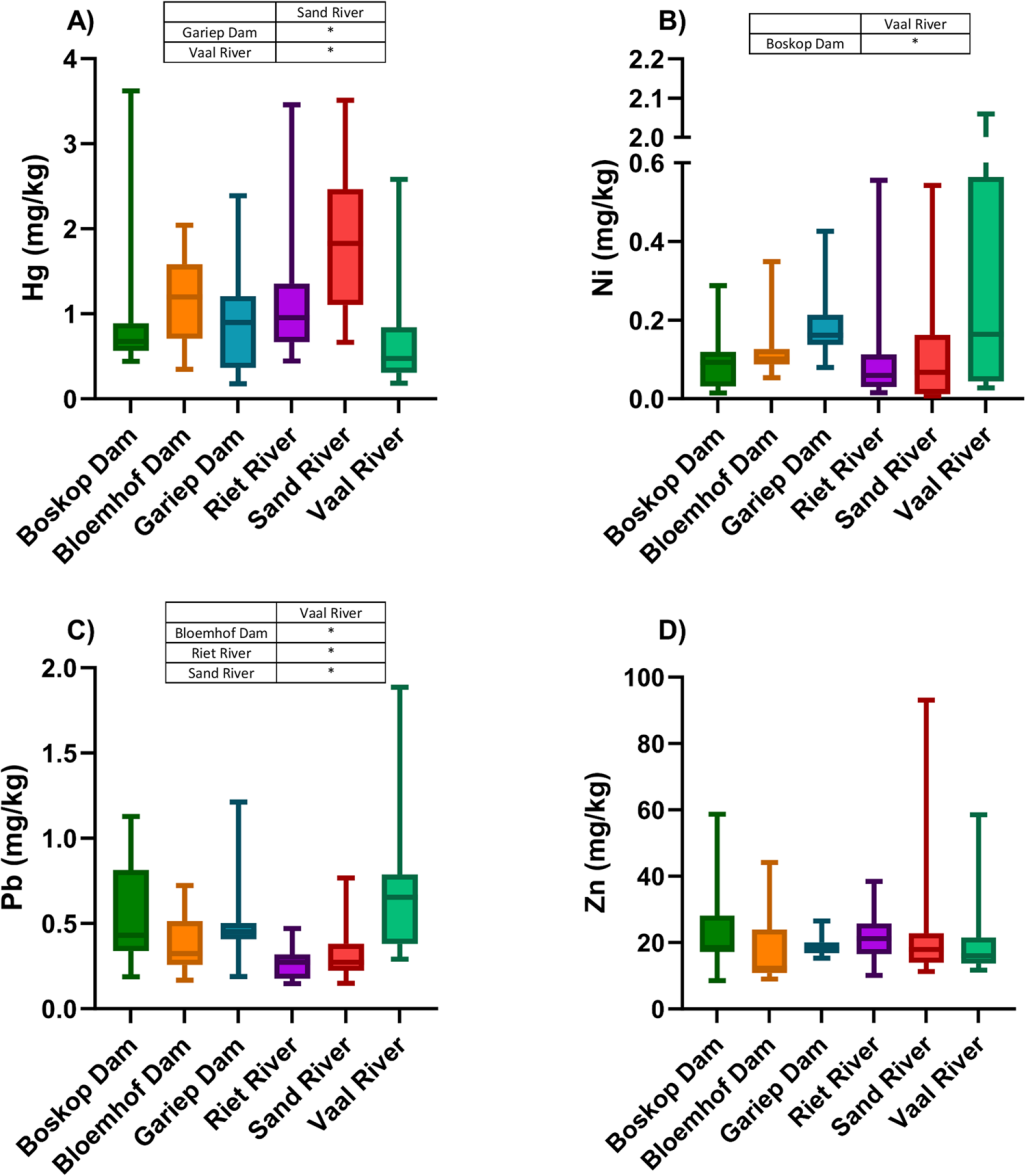
Box and whisker plots of the concentrations (mg/kg d.w.) of **A** Hg, **B** Ni, **C** Pb, and **D** Zn in *Clarias gariepinus* from the six sampling sites in the Orange-Vaal River basin. Mean concentrations are indicated with a solid line and the whiskers indicates the 5^th^ and 95^th^ percentiles. Significant differences (*p*< 0.05) between sites are indicated in table format by means of an asterisk

The concentrations of Hg and Zn were the highest in *C. gariepinus* from the Sand River, and concentrations of Hg were significantly higher compared to *C. gariepinus* from the Gariep Dam and the Vaal River (Fig. 3A, D). The *C. gariepinus* from the Vaal River had the highest concentrations of Ni and Pb that were significantly higher compared to Boskop Dam, as well as Bloemhof Dam, Riet River, and Sand River, respectively (Fig. 3B, C).

**Fig. 4:**
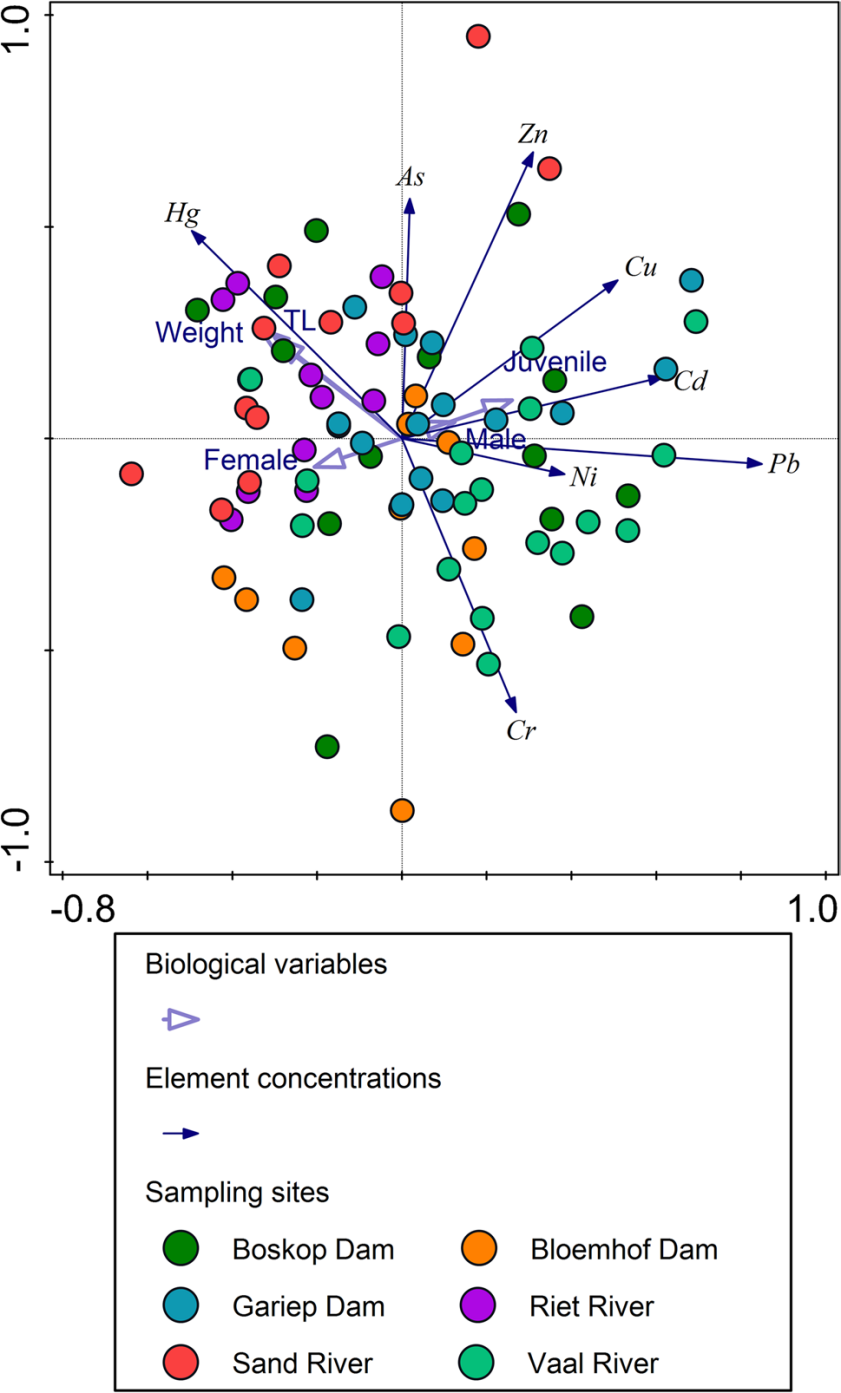
A PCA biplot of the element concentrations in *Clarias gariepinus* from six sampling sites in the Orange-Vaal River basin, with the biological variables overlayed as supplementary variables. The total variation explained is 44.1%, with 24.0% on the first axis and 20.1% on the second axis

From the PCA biplot, it is evident that larger *C. gariepinus* accumulated higher concentrations of Hg (especially at Boskop Dam, Riet River, and Sand River), while *C. gariepinus* from Boskop Dam and the Vaal River was associated with higher concentrations of Cd, Cr, Cu, Ni, and Pb (Fig. 4). Interestingly, female *C. gariepinus* showed lower element concentrations compared to males and juveniles.

### Human health risks

Carcinogenic and non-carcinogenic human health risks were assessed by calculating the CR and HQ from the EDI and ADD, in addition to the CSF and reference dose (RfD), respectively. An unacceptable carcinogenic health risk with CR values > 10^−4^ was found at the six sites for As and Cr. Fish from the Gariep Dam had the highest CR value for As of 75 and the lowest at Bloemhof Dam (21), while Bloemhof Dam had the highest CR value for Cr of 7 (Table 3). Furthermore, for all six sites, most of the elements of interest had HQ < 1 values, except for As and Hg. The HQ values for As ranged from 1.5 at Bloemhof Dam to 5.6 at Gariep Dam, while for Hg, the HQ values ranged between 1.8 at the Vaal River and 5.1 at the Sand River (Table 2). These HQ values indicate that both As and Hg concentrations pose a high probability of non-carcinogenic adverse health effects associated with regular consumption of *C. gariepinus* from the Orange-Vaal River basin.

**Table 2:** Mean cancer risk (CR) and hazard quotients (HQs), as well as the maximum safe consumption limit per day for *Clarias gariepinus* from six sampling sites in the Orange-Vaal River basin, calculated on the mean trace element concentration in muscle tissue. The human health risks were calculated with the assumption that a person of 60 kg consumes one fish meal (150 g) twice a week for HQ or daily for CR. Most of the HQ and CR values investigated were far below one, and only values of HQ > 1, indicating a high probability of adverse health effects, as well as CR > 10^−4^, indicating an unacceptable risk to humans that consume these fish, are indicated in this table

Elements	Boskop Dam	Bloemhof Dam	Gariep Dam	Riet River	Sand River	Vaal River
Cancer risk (CR) (× 10^−4^)						
As	46	21	75	49	54	39
Cr	3	7	3	1	1	3
Hazard quotients for non-carcinogenic risks (HQ)						
As	3.4	1.5	5.6	3.6	4.0	2.9
Hg	3.0	3.1	2.5	3.3	5.1	1.8
Maximum safe consumption limit per day (g)						
As (HQ)	32.4	14.6	9.0	13.9	12.5	17.4
As (CR)	73.2	162.0	44.8	69.4	62.6	87.2
Cr	133.5	56.3	119.6	352.0	440.0	108.5
Hg	16.1	16.7	19.7	15.0	9.8	28.4

Cancer slope factors (mg/kg/day): As (1.5); Cd (0.001); Cr (0.5); Ni (0.84) (USEPA 2000; IRIS 2017).

Reference dose (RfD) (μg/kg): As (0.3); Cd (1); Cr (3); Cu (40); Hg (0.1); Ni (20); Zn (300) (USEPA 2005).

## Discussion

**Fig. 5:**
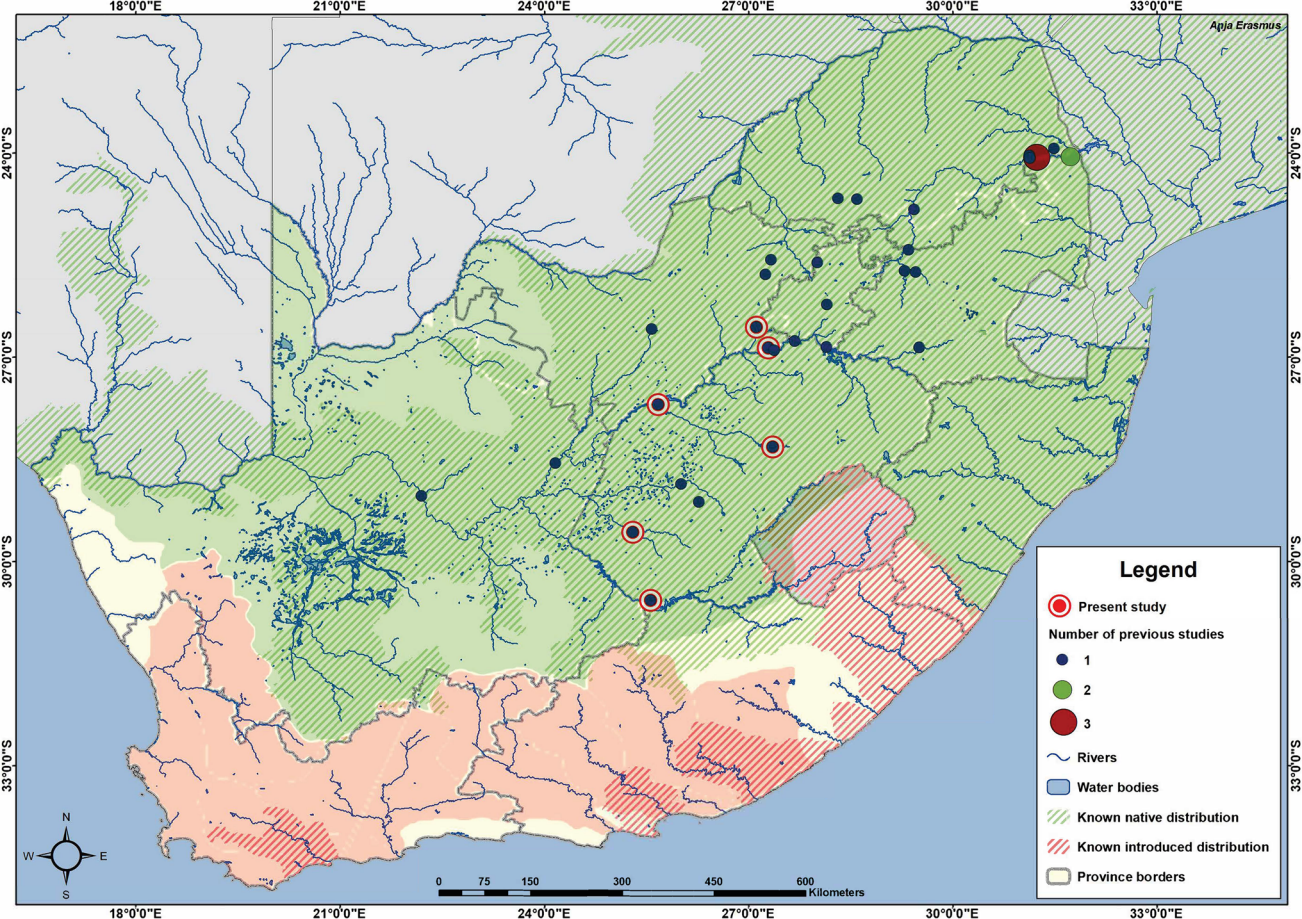
Map indicating the number of studies on element accumulation in *Clarias gariepinus* in South Africa. The sites in the present study are indicated with a red outline, while the sampling sites of previous studies are indicated based on the number of studies completed per locality ranging from one to three studies per site. The known reported distribution of *C. gariepinus* is indicated with striped overlays (IUCN 2019), while distribution ranges are indicated in shaded overlays (Picker and Griffiths 2011). Green overlays indicate the native distribution, while red overlays indicate the introduced or translocated distribution of *C. gariepinus*

In South Africa, 17 previous studies have been completed on the bioaccumulation of elements in the widely distributed *C. gariepinus* from the late 1980s until recently, covering 24 different localities (Table 3; Fig. 5). Several studies concluded that *C. gariepinus* is a good bioaccumulation indicator species due to its feeding habits, association with the sediment, and occupying higher trophic levels in aquatic ecosystems indicating that biomagnification of elements occur (Table 3). Of the 17 previous studies, only five included an assessment of human health risks posed by the consumption of *C. gariepinus* contaminated with high element concentrations and found that elements such as As, Cr, Hg, and Ni posed human health risks, irrespective of different sampling locations (van der Heever and Frey 1994; van der Heever and Frey 1996; Jooste et al. 2015; Marr et al. 2015; Erasmus et al. 2022b). In the study by Erasmus et al. (2022b), the concentrations of As, Cr, and Ni posed human health risks associated with the consumption of specifically *C. gariepinus*. These elements also exceeded the levels of concern for sediment dwelling organisms based on the consensus-based sediment quality guidelines (CBSQG); thus, it can be seen that *C. gariepinus* represents the state of the aquatic ecosystems in which they are found.

**Table 3:** Comparison of present and previous studies conducted on element concentrations in *Clarias gariepinus* from sampling sites in South Africa. The localities, sampling period, number of individuals, mean and standard deviation of the total length or range in parenthesis, mean and standard deviation of the weight or range in parenthesis, and mean and standard deviation of the element concentrations in milligrams per kilogram dry weight, as well as the references, are shown

Location	Time period	*n*	Total length (cm)	Weight (kg)	As	Cd	Cr	Cu	Hg	Ni	Pb	Zn	Reference
Boskop Dam (Mooi River)	2018–2020	15	59 (34–80)	1.7 (0.36–3.6)	3.8 ± 1.6	0.038 ± 0.024	0.69 ± 0.79	1.5 ± 1.4	1.1 ± 0.97	0.091 ± 0.069	0.55 ± 0.29	23 ± 12	Present study
Bloemhof Dam (Vaal River)	2018–2020	10	105 (75–165)	4.4 (2.92–6.9)	1.7 ± 0.61	0.023 ± 0.012	1.7 ± 1.2	2.0 ± 2.1	1.2 ± 0.55	0.12 ± 0.083	0.37 ± 0.17	18 ± 11	Present study
Gariep Dam (Orange River)	2018–2020	15	74 (38–115)	3.4 (0.38–12)	6.2 ± 2.3	0.052 ± 0.033	0.77 ± 0.52	1.2 ± 0.57	0.94 ± 0.67	0.18 ± 0.081	0.53 ± 0.28	19 ± 3.4	Present study
Riet River	2018–2020	12	79 (37–120)	4.2 (0.40–13)	4.0 ± 1.0	0.024 ± 0.015	0.26 ± 0.11	0.83 ± 0.33	1.2 ± 0.90	0.11 ± 0.15	0.27 ± 0.10	22 ± 7.5	Present study
Sand River	2018–2020	12	72 (49–87)	2.9 (1.14–5.1)	4.4 ± 1.3	0.036 ± 0.027	0.21 ± 0.037	1.4 ± 1.5	1.9 ± 0.87	0.12 ± 0.15	0.33 ± 0.17	24 ± 22	Present study
Vaal River	2018–2020	18	57 (34–80)	1.9 (0.28–4.7)	3.2 ± 1.6	0.042 ± 0.026	0.85 ± 0.94	1.1 ± 0.44	0.65 ± 0.56	0.41 ± 0.59	0.70 ± 0.38	21 ± 12	Present study
Germiston Lake	1988–1989	11	(46–77)	(0.68–3.6)	-	-	-	9.0 ± 2.6	-	-	-	59 ± 32	Bezuidenhout et al. 1990
Bloemspruit sewage works	1990	30	-	-	-	-	0.62 ± 0.19	62 ± 16	1.5 ± 0.22	-	-	618 ± 108	van der Heever and Frey 1994; van der Heever and Frey 1996
Krugersdrift Dam (Modder River)	1990	30	-	-	-	-	0.46 ± 0.12	31 ± 9.3	0.96 ± 0.06	-	-	1 082 ± 56	van der Heever and Frey 1994; van der Heever and Frey 1996
Selati River	1990–1991	38	47 ± 28	1.0 ± 0.62	-	-	102 ± 9.2	12 ± 8.1	-	45 ± 4.7	84 ± 9.1	68 ± 37	du Preez et al. 1997
Olifants River (Mamba)	1990–1091	62	53 ± 40	1.2 ± 0.84	-	-	82 ± 18	9.3 ± 0.75	-	43 ± 7.2	79 ± 11	56 ± 25	du Preez et al. 1997
Olifants River (Balule)	1990–1991	56	50 ± 30	1.4 ± 0.61	-	-	64 ± 7.3	8.0 ± 2.2	-	46 ± 4.7	65 ± 8.7	62 ± 28	du Preez et al. 1997
Olifants River (Mamba)	1994	77	-	-	-	-	128 ± 52	18 ± 7.0	-	73 ± 29	-	-	Avenant-Oldewage and Marx 2000a, 2000b
Olifants River (Balule)	1994	80	-	-	-	-	124 ± 31	9.0 ± 2.7	-	71 ± 19	-	-	Avenant-Oldewage and Marx 2000a, 2000b
Loskop Dam (Olifants River)	1994–1995	55	(12 – 95)	(0.17 – 7.3)	-	-	-	2.0 ± 2.0	-	-	-	35 ± 27	Kotze et al. 1999
Olifants River (Mamba)	1994–1995	89	(30–87)	(0.28–6.5)	-	-	-	2.0 ± 1.0	-	-	-	31 ± 12	Kotze et al. 1999
Nhlanganini Dam (Nhlanganini River)	1994–1995	17	(37–73)	(0.35–4.8)	-	-	-	1.0 ± 1.7	-	-	-	46 ± 26	Kotze et al. 1999
Klein Olifants River	1994–1995	41	(40–69)	(0.20–2.8)	-	-	31 ± 13	7.0 ± 3.7	-	16 ± 8.5	7.5 ± 2.3	44 ± 12	Coetzee et al. 2002
Olifants River	1994–1995	80	(40–69)	(0.20–2.8)	-	-	24 ± 8.4	5.9 ± 2.5	-	15 ± 6.6	5.4 ± 1.7	43 ± 11	Coetzee et al. 2002
Vaal Barrage	1998–2000	100	-	-	-	-	0.58 ± 0.73	2.80 ± 1.57	-	2.48 ± 2.87	4.20 ± 2.58	26 ± 11	Crafford and Avenant-Oldewage 2010, 2011
Vaal Dam	1998–2000	110	-	-	-	-	0.37 ± 0.66	2.87 ± 4.06	-	1.91 ± 2.44	3.05 ± 2.17	40 ± 17	Crafford and Avenant-Oldewage 2010, 2011
Medunsa Lake	2010	10	-	0.09 ± 0.003	-	-	3.9 ± 0.65	0.65 ± 0.01	-	3.34 ± 0.32	0.12 ± 0.01	17 ± 0.12	Olowoyo et al. 2011
Flag Boshielo Dam (Olifants River)	2010	10	-	-	0.93 ± 0.31	-	30 ± 5.6	4.3 ± 2.8	-	1.9 ± 1.2	5.3 ± 1.6	20 ± 12	Jooste et al. 2015; Marr et al. 2015
Phalaborwa Barrage (Olifants River)	2010	13	-	-	0.62 ± 1.6	-	11 ± 1.2	3.1 ± 0.62	-	0.93 ± 0.62	1.2 ± 0.62	154 ± 37	Jooste et al. 2015; Marr et al. 2015
Upper Vaal River	2010	10	(63–93)	4.0	0.06 ± 0.01	0.02 ± 0.01	0.29 ± 0.03	0.05 ± 0.03	0.03 ± 0.02	0.03 ± 0.00	0.01 ± 0.00	0.55 ± 0.18	Pheiffer et al. 2014
Middle Vaal River	2010	10	(66–77)	4.1	0.05 ± 0.00	0.05 ± 0.01	0.25 ± 0.01	0.05 ± 0.01	0.05 ± 0.01	0.03 ± 0.00	0.01 ± 0.00	0.69 ± 0.17	Pheiffer et al. 2014
Lower Vaal River	2010	10	(67–107)	6.0	0.06 ± 0.00	0.03 ± 0.00	0.27 ± 0.01	0.04 ± 0.01	0.04 ± 0.00	0.03 ± 0.00	0.01 ± 0.00	0.63 ± 0.17	Pheiffer et al. 2014
Orange River	2010	10	(53–72)	2.4	0.05 ± 0.01	0.25 ± 0.26	0.21 ± 0.03	0.06 ± 0.34	0.73 ± 0.02	0.03 ± 0.01	0.02 ± 0.01	0.66 ± 0.14	Pheiffer et al. 2014
Klein Nyl River	2012	5	-	-	-	-	2.1 ± 1.1	16 ± 3.5	-	-	-	48 ± 9.3	Musa et al. 2017
Nyl River	2012	5	-	-	-	-	2.1 ± 0.67	6.1 ± 1.3	-	-	-	72 ± 19	Musa et al. 2017
Baberspan (Harts River)	2014	10	50 ± 97	1.1 ± 6.6	0.11 ± 0.09	-	3.9 ± 7.3	5.6 ± 4.3	0.49 ± 0.34	0.59 ± 0.28	0.26 ± 0.21	31 ± 28	Malherbe et al. 2015
Olifantsnek Dam (Hex River)	2017–2018	20	53 ± 15	1.1 ± 0.58	3.5 ± 2.0	0.09 ± 0.08	1.3 ± 0.41	1.4 ± 0.53	-	0.44 ± 0.33	1.1 ± 0.71	20 ± 6.4	Erasmus et al. 2022b
Bospoort Dam (Hex River)	2017–2018	16	73 ± 13	3.3 ± 1.7	7.9 ± 2.8	0.14 ± 0.13	2.2 ± 1.3	1.4 ± 0.46	-	0.34 ± 0.19	1.9 ± 1.3	18 ± 6.3	Erasmus et al. 2022b

Comparing the present results to the results obtained in 2010 in the Orange-Vaal River basin (Pheiffer et al. 2014), most of the element concentrations were higher with the only exception of Cd that was within the same range (Table 3). This indicates that water quality and ecosystem services and functions have decreased in the last 8 years. This is important to note, as the water quality and ecosystem health of South African rivers are constantly under pressure and decreasing with time, indicating that monitoring programs and legislation must be improved to ensure the preservation of a scarce natural resource. When comparing the data with previous studies completed on the element concentrations in *C. gariepinus*, it clearly indicates that there is a bias to sampling locations in the north-eastern part of South Africa, only assessing the bioaccumulation of elements in the native distribution of *C. gariepinus* (Fig. 5). It would be interesting to assess the bioaccumulation of elements in the introduced distribution of *C. gariepinus*, as most of the river systems in the introduced region are not as affected by anthropogenic activities as in the native distribution range, and whether they still reflect the ecosystem condition at these localities. The only locations where more than one study was conducted was at Mamba weir and Balule weir, which do not really contribute any progressive data on human health risk associated with consumption, as both sites are located in Kruger National Park (KNP) and no known local community uses catfish as food source in this protected area. However, these localities provide important accumulation data as most of the rivers that flow through the KNP are impacted by several anthropogenic activities in their upper catchments.

### Element bioaccumulation in *Clarias gariepinus*

The bioaccumulation of elements in the Orange-Vaal River basin is quite site specific, where the Gariep Dam had the highest concentrations of As and Cd, Bloemhof Dam had the highest concentrations of Cr and Cu, the Sand River had the highest concentrations of Hg and Zn, while the Vaal River had the highest concentrations of Ni and Pb. Although all of these elements can enter the aquatic ecosystem via natural weathering of element-rich minerals and the surrounding geology, various anthropogenic activities including different land uses might be potential sources of pollution. Both As and Cd are normally associated with various mining activities, as well as metal processing and smelting processes; these anthropogenic activities do not occur in the vicinity or in the upper catchment of the Gariep Dam; however, both of these elements are associated with pesticides and fertilizers that can enter the Gariep Dam through runoff from intensive agricultural land-use activities in the upper catchment (Jang et al. 2016; Friberg 2018). Chromium can enter the aquatic environment via various industrial production activities (metal, leather, dyes, paints, ceramics, explosives, glass, and paper), but also via cooling waters for industrial and urban uses, where it is used as a corrosion inhibitor (Zhitkovich 2011). Copper can originate from corrosion of brass and copper pipes by acidic waters, mining activities, smelting and refining industries, and sewage effluent, as well as runoff from agricultural activities using fungicides and pesticides (Shrivastava 2009). All of the above-mentioned anthropogenic and land-use activities occur in the upper catchments (Sand and Vaal Rivers) that drain into Bloemhof Dam. Sources of Hg are normally associated with formal gold mining activities, while artisanal gold miners use Hg to wash sediments to form amalgam (Hg combined with gold pieces), which is blowtorched to burn off the Hg and leave the gold pieces. These artisanal activities are responsible for 35% of all Hg pollution released into the environment (UNEP 2023); however, the main source of Hg contamination in South Africa is the combustion of fossil fuels and aerial deposition from coal-fired power plants (Walters et al. 2011; Erasmus et al. 2022a). Sources of Zn include industrial and mining activities and sewage effluent, as well as runoff from agricultural activities using fertilizers and insecticides (Callender 2000); anthropogenic sources for both Hg and Zn occur in the Sand River and this sampling location is also located in one of the main aerial transport pathways from the Mpumalanga Highveld where most of the coal-fired power plants of South Africa are situated (Erasmus et al. 2022a; van Rooyen et al. 2023). The Vaal River has been described as one of Africa’s work horse rivers, receiving runoff and sewage effluent from three major metropolitan cities, approximately 13,600 wet industries, as well as several gold mines (Wepener et al. 2011). Anthropogenic sources of Ni originate from various industrial and commercial activities (metallurgical, chemical, and food processing), as well as sewage effluent (Cempel and Nikel 2006), while Pb can enter the aquatic ecosystem by industrial and municipal wastewater discharge, milling, and smelting of metals, as well as combustion of fossil fuels (Obeng-Gyasi 2019). It is noteworthy that one of South Africa’s largest metallurgical industries is located in the upper Vaal River and has been operational since 1947 (ArcelorMittal 2023). Therefore, the land-use and anthropogenic activities located in the vicinity or in the upper catchment of the four sites with the highest element concentrations found and accumulated in *C. gariepinus* indicate that this fish species represents the environmental health of aquatic ecosystems.

### Human health risks associated with the consumption of *Clarias gariepinus*

There are several local communities that practice small-scale fisheries and subsistence fishing within the Orange-Vaal River basin and are reliant on fish as protein source, especially *C. gariepinus*. However, there is no information on actual fish consumption available in the Orange-Vaal River basin; therefore, an estimated human health risk assessment was performed. A conservative risk assessment was used and considered adults of 60 kg consuming a 150-g portion of fish twice a week (Heath et al. 2004). Individuals who are more sensitive and susceptible to chronic exposure to toxic elements include pregnant women, lactating mothers, their infants, and children (Javed and Usmani 2019). With the approved National Freshwater (Inland) Wild Capture Fisheries Policy in 2021, more communities will utilize freshwater ecosystem services like fish, while the government is proposing large-scale fisheries in large impoundments like the Gariep Dam.

The results of the CR indicated that both As and Cr exceeded the unacceptable risk associated with fish consumption. The highest risk from As was at the Gariep Dam, while the lowest was at Bloemhof Dam, while for Cr, the highest risk was at Bloemhof Dam and the lowest risk was at the Riet and Sand Rivers. According to the South African National Cancer Registry, the background cancer risk rates are approximately 1250 out of 10,000 for men and 1110 out of 10,000 for women (CANSA 2015). Thus, the CR of 75 out of 10,000 for As at Gariep Dam is well below the background cancer risk. However, the added CR associated with fish consumption in addition to the already high background cancer risk can have an accumulation effect. The only study to assess carcinogenic risks in *C. gariepinus* in South Africa was carried out by Erasmus et al. (2022b). They found that As also posed a high CR of 93 out of 10,000 from an impoundment that is impacted by intensive platinum mining activities, where the CR of Cr was 10 out of 10,000 which is more or less in the same range as Bloemhof Dam from the present study. From the calculations of the maximum safe consumption limit, it indicates that the consumption of *C. gariepinus* from the Sand River is the safest, where a person of 60 kg can consume on average 131 g of fish portion twice a week based on all of the potential toxic elements, while the consumption of fish from the Gariep Dam poses the highest risk with a safe consumption average of 48 g of fish portion twice a week. This is a major concern as *C. gariepinus* is used internationally as an aquacultural species that accounts for approximately 2% of the global production (Anchor Environmental 2012; FAO 2013). Global production of *C. gariepinus* increased from 2000 to approximately 200,000 t from 2000 until 2010 (Anchor Environmental 2012; FAO 2013). This shows that this is not only a local problem in South Africa but can also pose an international human health risk.

From the results of the human health risk assessment, the concentrations of As and Hg exceeded the safe human consumption levels and can pose non-carcinogenic risks. Although only these two elements exceeded the HQ values, it is important to remember that the risk assessment was only based on single-element exposure. However, these fish are exposed to multiple pollutants (elements, as well as organic contaminants) and may potentially pose an even greater risk than assessed. From the previous studies that completed human health assessments, elements such as As, Cr, Co, Hg, and Sb posed non-carcinogenic risks (van der Heever and Frey 1994; van der Heever and Frey 1996; Jooste et al. 2015; Marr et al. 2015; Erasmus et al. 2022b).

### Linking animal, environmental, and human health within the One Health concept

The bioaccumulation of elements in *C. gariepinus* clearly indicated that they represent the ecosystem health of the aquatic ecosystems they were collected from, and it reflects the land-use activities and anthropogenic stressors at each location. Other studies also found that the elements that exceeded the CBSQG were also the elements that posed human health risks associated with the consumption of contaminated fish. Although the element concentrations in *C. gariepinus* collected at the six sites in the Orange-Vaal River basin represent higher concentrations compared to the study completed in 2010 (Pheiffer et al. 2014), no impaired fish health was observed, indicating that these species have a high tolerance to extreme environments and can be used as a good accumulation species as it will still occur in highly polluted ecosystems, rather than the more sensitive species. There were both carcinogenic and non-carcinogenic human health risks associated with the consumption of *C. gariepinus* from various sites in the present study and can be elaborated on an international scale. The present study therefore links the animal, environmental, and human health and indicates the inseparable links between these different factors.

### Broader ecological impacts and policy implications

Potential toxic elements enter aquatic ecosystems from various anthropogenic sources and do not only affect specific species that can accumulate these elements but can also have an effect on community structures or even cause alterations in organism well-being. Macroinvertebrates, like other aquatic biota, have different tolerances and body burdens towards elements and can either be eliminated from community structures (in the case of sensitive taxa) or become dominant in the community structure as competition for resources is declining (in the case of tolerant taxa) (Bervoets et al. 2016). A study by Erasmus et al. (2021), focusing on element concentrations from platinum mining activities, found that these anthropogenic stressors affect the macroinvertebrate community structure. Sensitive taxa significantly declined from reference condition towards impacted conditions, while tolerant taxa increased in numbers and dominated at heavily impacted sites reducing the overall species diversity. Element contaminated soils and sediments have also been proven to affect reproduction and growth in *Caenorhabditis elegans* (a free-living nematode) (Díaz-Morales et al. 2021), while in *Cyprinus carpio* (common carp), increased element concentrations caused a decrease in reduced glutathione content and an increase in lipid peroxidation (Erasmus et al. 2020b).

The use of the One Health approach and using *C gariepinus* to monitor and assess element concentrations in South Africa’s largest river basin proved valuable in assessing the influences of multiple anthropogenic stressors on element bioaccumulation and how it not only poses a risk to ecosystem function and health but to human health too. It is recommended that more frequent element monitoring and assessment programs should be implemented in various river systems across South Africa. These programs should assess and monitor different fish species that are frequently targeted by subsistence fishers, especially with the National Freshwater (Inland) Wild Capture Fisheries Policy that was approved in 2021. Research on the implementation of these monitoring and assessment of element concentrations into integrated water resource management, resource quality objectives, environmental flows, and aquatic ecosystem monitoring programs are also needed in South Africa to enhance the management of scarce freshwater resources and the aquatic biota living in these systems.

## Conclusions

Previous studies mainly only focused on assessing element concentrations in environmental matrices (water and sediment) and reporting concentrations in fish species. The One Health concept is an integrated approach to assess and connect the important interactions between environmental, animal, and human health. The present study assessed the element concentrations from six sites within the large distribution range of *C. gariepinus* and found that the element concentrations accumulated correspond to the anthropogenic land-use activities associated with each site. It also assessed the carcinogenic and non-carcinogenic human health risks associated with the consumption of contaminated *C. gariepinus*, while integrating the important link between environmental, animal and human health. It is evident that there is a bias in South Africa on where element concentrations in *C. gariepinus* are reported and that there are no studies that assessed the contamination in *C. gariepinus* within its introduced distribution range. As human health risks were evidently associated with the consumption of *C. gariepinus*, it has a local, national, and international relevance as *C. gariepinus* are used as an aquaculture species. This study, therefore, highlights the importance of monitoring toxic pollutants and their human health effects associated with the consumption of contaminated fish, while also linking the interaction between environmental, animal, and human health.

## Declarations

### Disclaimer

Opinions, findings, conclusions, and recommendations expressed in this publication are those of the authors, and the NRF, SAIAB and NWU accepts no liability whatsoever in this regard.

## References

[CR1] ArcelorMittal, 2023. Company history. https://arcelormittalsa.com/whoweare/ourhistory.aspx (Accessed 15 Nov 2022).

[CR2] Avenant-Oldewage A, Marx HM (2000). Bioaccumulation of chromium, copper and iron in the organs and tissues of *Clarias gariepinus* in the Olifants River, Kruger National Park. Water SA.

[CR3] Avenant-Oldewage A, Marx HM (2000). Manganese, nickel and strontium bioaccumulation in the tissues of the African sharptooth catfish, *Clarias gariepinus* from the Olifants River, Kruger National Park. Koedoe.

[CR4] Barbour MT, Gerritsen J, Snyder BD, Stribling JB (1999). Rapid Bioassement Protocol for use in streams and wadeable rivers: periphyton, bentic macroinvertebrates and fish, second ed.

[CR5] Bervoets L, De Jonge M, Blust R (2016). Identification of threshold body burdens of metals for the protection of the aquatic ecological status using two benthic invertebrates. Environ Pollut.

[CR6] Bezuidenhout LM, Schoonbee HJ, de Wet LPD (1990). Heavy metal content in organs of the African sharptooth catfish, *Clarias gariepinus* (Burchell), from a Transvaal lake affected by min and industrial effluents. Part 1. Zinc and copper. Water SA.

[CR7] Callender E (2000). The urban environmental gradient: anthropogenic influences on the spatial and temporal distributions of lead and zinc in sediments. Environ Sci Technol.

[CR8] Cancer Association of South Africa (CANSA), 2015. Fact sheet on the top ten cancers per population group. http://www.cansa.org.za/files/2015/09/Fact-Sheet-Top-Ten-Cancersper-Population-Group-Sept-2015.pdf (Accessed 3 Sept. 2022).

[CR9] Cempel M, Nikel G (2006). Nickel: a review of its sources and environmental toxicology. Pol J Environ Stud.

[CR10] Coetzee L, du Preez HH, van Vuren JHJ (2002). Metal concentrations in *Clarias gariepinus* and *Labeo umbratus* from the Olifants and Klein Olifants River, Mpumalanga, South Africa: zinc, copper, manganese, lead, chromium, nickel, aluminium and iron. Water SA.

[CR11] Crafford D, Avenant-Oldewage A (2010). Bioaccumulation of non-essential trace metals in tissues and organs of *Clarias gariepinus* (sharptooth catfish) from the Vaal River system – strontium, aluminium, lead and nickel. Water SA.

[CR12] Crafford D, Avenant-Oldewage A (2011). Uptake of selected metals in tissues and organs of *Clarias gariepinus* (sharptooth catfish) from the Vaal River system – chromium, copper, iron manganese and zinc. Water SA.

[CR13] Department of Forestry, Fisheries and the Environment (DFFE), 2021. National Freshwater (Inland) Wild Capture Fisheries Policy for South Africa. Department of Forestry, Fisheries and the Environment, Pretoria. https://www.dffe.gov.za/sites/default/files/legislations/wildcapturefisheries policy.pdf (Accessed 22 Sept. 2022).

[CR14] Department of Statistics South Africa (Stats SA), 2017. Four facts about our rivers you probably didn’t know. https://www.statssa.gov.za/?p=9490 (Accessed 22 Feb. 2023).

[CR15] Department of Water and Sanitation (DWS) (2019). State of river report: river ecostatus monitoring programme 2017-2018.

[CR16] Díaz-Morales DM, Erasmus JH, Bosch S, Nachev M, Smit NJ, Zimmermann S, Wepener V, Sures B (2021). Metal contamination and toxicity of soils and river sediments from the world’s largest platinum mining area. Environ Pollut.

[CR17] du Preez HH, van der Merwe M, van Vuren JHJ (1997). Bio-accumulation of selected metals in African sharptooth catfish *Clarias gariepinus* from the lower Olifants River, Mpumalanga, South Africa. Koedoe.

[CR18] Environmental A (2012). African sharptooth catfish *Clarias gariepinus*. DAFF Biodiversity Risk- and Benefit Assessment (BRBA) of alien species in aqua-culture in South Africa.

[CR19] Erasmus JH, Lorenz AW, Zimmermann S, Wepener V, Sures B, Smit NJ, Malherbe W (2021). A diversity and functional approach to evaluate the macroinvertebrate responses to multiple stressors in a small subtropical austral river. Ecol Indic.

[CR20] Erasmus JH, Malherbe W, Weyl OLF, Sures B, Wepener V, Smit NJ (2019). First record of *Labeo capensis* (Smith, 1841) in the Crocodile River (West) system: another successful non-native freshwater fish introduction in South Africa. Afr J Aquat Sci.

[CR21] Erasmus JH, Malherbe W, Zimmermann S, Lorenz AW, Nachev M, Wepener V, Sures B, Smit NJ (2020). Metal accumulation in riverine macroinvertebrates from a platinum mining region. Sci Total Environ.

[CR22] Erasmus JH, Smit NJ, Gerber R, Schaeffner BC, Nkabi N, Wepener V (2022). Total mercury concentrations in sharks, skates and rays along the South African coast. Mar Pollut Bull.

[CR23] Erasmus JH, Wepener V, Nachev M, Zimmermann S, Malherbe W, Sures B, Smit NJ (2020). The role of fish helminth parasites in monitoring metal pollution in aquatic ecosystems: a case study in the world’s most productive platinum mining region. Parasitol Res.

[CR24] Erasmus JH, Zimmermann S, Smit NJ, Malherbe W, Nachev M, Sures B, Wepener V (2022). Human health risks associated with consumption of fish contaminated with trace elements from intensive mining activities in a peri-urban region. Sci Total Environ.

[CR25] Essack SY (2018). Environment: the neglected component of the One Health triad. Lancet Planet Health.

[CR26] FAO (2013). Cultured aquatic species information programme: *Clarias gariepinus* (Burchell, 1822).

[CR27] Friberg L (2018). Cadmium in the environment.

[CR28] Froese, R., Pauly, D., 2023. FishBase: *Clarias gariepinus*. https://www.fishbase.se/summary/Clarias-gariepinus.html (Accessed 22 Feb 2023).

[CR29] Heath RGM, du Preez HH, Genthe B, Avenant-Oldewage A (2004). Freshwater fish and human health. Reference Guide. WRC Report No. TT212/04.

[CR30] Integrated Risk Information System (IRIS), 2017. IRIS assessments of United States Environmental Protection Agency. https://iris.epa.gov/AtoZ/?list_type=alpha (Accessed 22 Sept. 2022).

[CR31] Jang YC, Somanna Y, Kim H (2016). Source, distribution, toxicity and remediation of arsenic in the environment – a review. Int J Appl Environ Sci.

[CR32] Javed M, Usmani N (2019). An overview of the adverse effects of heavy metal contamination on fish health. Proc. Natl. Acad. Sci., India. Sect B.

[CR33] Jooste A, Marr SM, Addo-Bediako A, Luus-Powell WJ (2015). Sharptooth catfish shows its metal: a case study of metal contamination at two impoundments in the Olifants River, Limpopo River system, South Africa. Ecotoxicol Environ Saf.

[CR34] Kawarazuka N, Béné C (2011). The potential role of small fish species in improving micronutrient deficiencies in developing countries: building evidence. Public Health Nutr.

[CR35] Kotze P, du Preez HH, van Vuren JHJ (1999). Bioaccumulation of copper and zinc in *Oreochromis mossambicus* and *Clarias gariepinus*, from the Olifants River, Mpumalanga, South Africa. Water SA.

[CR36] Mackenzie JS, Jeggo M, Daszak P, Richt JA (2013) One Health: the human-animal-environment interfaces in emerging infectious diseases. The concept and examples of a One Health approach. Springer, Berlin

[CR37] Malherbe, W., Beukes, J., Smit, N.J., 2015. Chromium, copper, nickel and zinc accumulation within selected fish species from a Ramsar site in Southern Africa. 7th International Toxicology Symposium in Africa, pp. 26–27.

[CR38] Marr SM, Jooste A, Addo-Bediako A, Luus-Powell WJ (2015). Are catfish from metal-polluted impoundments in the Olifants River, South Africa, safe for human consumption?. Inland Waters.

[CR39] Multisanti CR, Merola C, Perugini M, Aliko V, Faggio C (2022). Sentinel species selection for monitoring microplastic pollution: a review on one health approach. Ecol Indic.

[CR40] Murtaugh MP, Steer CJ, Sreevatsan S, Patterson N, Kennedy S, Sriramarao P (2017). The science behind One Health: at the interface of humans, animals, and the environment. Ann N Y Acad Sci.

[CR41] Musa R, Gerber R, Greenfield R (2017). A multivariate analysis of metal concentrations in two fish species of the Nyl River system, Limpopo province, South Africa. Bull Environ Contam Toxicol.

[CR42] Musoke D, Ndejjo R, Atusingwize E, Halage AA (2016). The role of environmental health in One Health: a Uganda perspective. One Health.

[CR43] Obeng-Gyasi E (2019). Sources of lead exposure in various countries. Rev Environ Health.

[CR44] Olowoyo JO, Mdakane STR, Okedey OO (2011). Comparing the levels of trace metal from two fish species harvested from treated waste water in Pretoria, South Africa. Pak J Biol Sci.

[CR45] Pheiffer W, Pieters R, van Dyk JC, Smit NJ (2014). Metal contamination of sediments and fish from the Vaal River, South Africa. Afr J Aquat Sci.

[CR46] Picker M, Griffiths C (2011). Alien & invasive animals: a South African perspective.

[CR47] Prata JC, da Costa JP, Lopes I, Andrady AL, Duarte AC, Rosha-Santos T (2021). A One Health perspective of the impacts of microplastics on animal, human and environmental health. Sci Total Environ.

[CR48] Sayer J, Cassman KG (2013). Agricultural innovation to protect the environment. Proc Natl Acad Sci USA.

[CR49] Selbach C, Mouritsen KN, Poulin R, Sures B, Smit NJ (2022). Bridging the gap: aquatic parasites in the One Health concept. Trends Parasitol.

[CR50] Shrivastava AK (2009). A review on copper pollution and its removal from water bodies by pollution control technologies. Indian J Environ Prot.

[CR51] Skelton P (2001) A complete guide to the freshwater fishes of southern Africa. Struik Nature, Cape Town

[CR52] The IUCN Red List of Threatened Species (IUCN), 2019. *Clarias gariepinus* (amended version of 2018 assessment, e.T166023A155051767). 10.2305/IUCN.UK.2018-2.RLTS.T166023A155051767.en. Accessed on 06 Apr 2023.

[CR53] United Nations Environment Programme (UNEP), 2023. Artisanal and small-scale gold mining (ASGM). https://www.unep.org/globalmercurypartnership/what-we-do/artisanal-and-small-scale-gold-mining-asgm (Accessed 15 Nov 2022).

[CR54] United States Environmental Protection Agency (USEPA) (2000). Guidance for assessing chemical contaminant data for use in fish advisories. Volume 2: Risk assessment and fish consumption limits. third ed. EPA 823-B-00–008.

[CR55] United States Environmental Protection Agency (USEPA), 2005. Guidelines for carcinogen risk assessment. https://www.epa.gov/sites/default/files/2013-09/documents/cancer_guidelines_final_3-25-05.pdf (Accessed 22 Sept. 2022).

[CR56] United States Environmental Protection Agency (USEPA) (2016). Development of national bioaccumulation factors: supplemental information for EPA;s 2015 human health criteria update.

[CR57] van As J, du Preez J, Brown L, Smit NJ (2013). The story of life & the environment.

[CR58] van der Heever DJ, Frey BJ (1994). Human health aspects of the metals zinc and copper in tissue of the African sharptooth catfish, *Clarias gariepinus*, kept in treated sewage effluent and the Krugersdrift Dam. Water SA.

[CR59] van der Heever DJ, Frey BJ (1996). Human health aspects of certain metals in tissue of the African sharptooth catfish, *Clarias gariepinus*, kept in treated sewage effluent and the Krugersdrift Dam: Chromium and mercury. Water SA.

[CR60] van Rooyen D, Erasmus JH, Gerber R, Nachev M, Sures B, Wepener V, Smit NJ (2023). Bioaccumulation and trophic transfer of total mercury through the aquatic food webs of an African sub-tropical wetland system. Sci Total Environ.

[CR61] Vergis J, Rawool DB, Malik SVS, Barbuddhe SB (2021). Food safety in fisheries: applications of One Health approach. Indian J Med Res.

[CR62] Walters CR, Somerset VS, Leaner JJ, Nel JM (2011). A review of mercury pollution in South Africa: current status. J Environ Sci Health A.

[CR63] Wepener V, van Dyk C, Bervoets L, O’Brien G, Covaci A, Cloete Y (2011). An assessment of the influence of multiple stressors on the Vaal River, South Africa. Phys Chem Earth.

[CR64] Weyl OLF, Barkhuizen L, Christison K, Dalu T, Hlungwani HA, Impson D, Sankar K, Mandrak NE, Marr SM, Sara JR, Smit NJ, Tweddle D, Vine NG, Wepener V, Zvavahera M, Cowx IG (2021). Ten research questions to support South Africa’s inland fisheries policy. Afr J Aquat Sci.

[CR65] Zhitkovich A (2011). Chromium in drinking water: sources, metabolism, and cancer risks. Chem Res Toxicol.

